# VariantHunter: a method and tool for fast detection of emerging SARS-CoV-2 variants

**DOI:** 10.1093/database/baad044

**Published:** 2023-07-06

**Authors:** Pietro Pinoli, Arif Canakoglu, Stefano Ceri, Matteo Chiara, Erika Ferrandi, Luca Minotti, Anna Bernasconi

**Affiliations:** Department of Electronics, Information, and Bioengineering, Politecnico di Milano, Via Ponzio 34/5, Milan 20133, Italy; Department of Anesthesia, Critical Care and Emergency, Fondazione IRCCS Ca’ Granda Ospedale Maggiore Policlinico, Via Francesco Sforza 28, Milan 20122, Italy; Department of Electronics, Information, and Bioengineering, Politecnico di Milano, Via Ponzio 34/5, Milan 20133, Italy; Department of Biosciences, University of Milan, Via Celoria 26, Milan 20133, Italy; Institute of Biomembranes, Bioenergetics and Molecular Biotechnology, Consiglio Nazionale delle Ricerche, via Amendola 122/O, Bari 70126, Italy; Department of Biosciences, University of Milan, Via Celoria 26, Milan 20133, Italy; Institute of Biomembranes, Bioenergetics and Molecular Biotechnology, Consiglio Nazionale delle Ricerche, via Amendola 122/O, Bari 70126, Italy; Department of Electronics, Information, and Bioengineering, Politecnico di Milano, Via Ponzio 34/5, Milan 20133, Italy; Department of Electronics, Information, and Bioengineering, Politecnico di Milano, Via Ponzio 34/5, Milan 20133, Italy

## Abstract

With the progression of the COVID-19 pandemic, large datasets of SARS-CoV-2 genome sequences were collected to closely monitor the evolution of the virus and identify the novel variants/strains. By analyzing genome sequencing data, health authorities can ‘hunt’ novel emerging variants of SARS-CoV-2 as early as possible, and then monitor their evolution and spread. We designed VariantHunter, a highly flexible and user-friendly tool for systematically monitoring the evolution of SARS-CoV-2 at global and regional levels. In VariantHunter, amino acid changes are analyzed over an interval of 4 weeks in an arbitrary geographical area (continent, country, or region); for every week in the interval, the prevalence is computed and changes are ranked based on their increase or decrease in prevalence. VariantHunter supports two main types of analysis: lineage-independent and lineage-specific. The former considers all the available data and aims to discover new viral variants. The latter evaluates specific lineages/viral variants to identify novel candidate designations (sub-lineages and sub-variants). Both analyses use simple statistics and visual representations (diffusion charts and heatmaps) to track viral evolution. A dataset explorer allows users to visualize available data and refine their selection. VariantHunter is a web application free to all users. The two types of supported analysis (lineage-independent and lineage-specific) allow user-friendly monitoring of the viral evolution, empowering genomic surveillance without requiring any computational background.

**Database URL** http://gmql.eu/variant_hunter/

## Introduction

At the beginning of 2023, the two largest collections of SARS-CoV-2 sequences, GISAID (1) and GenBank (2), respectively, contain 15.6 and 7.4 million entries; numbers are increasing as new sequences are continuously deposited. Genomics surveillance represents the first line of defense against the spreading of novel viral variants. Regional and national health authorities need to *hunt* novel emerging variants as early as possible, and then monitor their evolution (e.g. differentiation in new sub-variants by the accumulation of mutations) and spread (by controlling their prevalence with respect to other variants). Careful monitoring of emerging variants is paramount in the fight against COVID-19 to identify variants potentially associated with increased infectivity and/or a reduction in vaccine efficiency. In addition, genomics surveillance is critical to check diagnostic and sequencing tools: some variants may lead to either false negatives (for some diagnosis kits) or low-quality output sequencing (for given protocols). Genomics surveillance does not require that every viral sequence be sequenced, as it is sufficient to monitor a large-enough population of sequences—both at the global and regional levels—for computing the statistics that model and predict the current and near-future status of the pandemics.

Here, we introduce VariantHunter (http://gmql.eu/variant_hunter/), a highly flexible and user-friendly tool for the systematic monitoring of the evolution of SARS-CoV-2 both at the global and regional levels. The tool manipulates large files containing metadata and mutations of SARS-CoV-2 sequences. At the time of writing, the open implementation works on more than 7.5 million sequences of 2520 distinct lineages, where the most represented in the database correspond to previously designated Variants of Concern (VOCs), namely B.1.1.7 (Alpha), AY.4 (Delta), BA.1/BA.1.1 (Omicron 1) and BA,2 (Omicron 2). Instead, the currently designated variant of interest XBB.1.5 has more than 62k sequences and other variants under monitoring are less represented (3). This dataset provides a broad representation of genomes collected in the United States and given European countries (especially, the United Kingdom, Germany and Denmark), whereas it holds less information on other countries—where sequence collection and deposition have been operated not continuously. Mutations are typically considered on 12 different proteins of SARS-CoV-2, but the most interesting mutations that characterize variants are usually located on the Spike protein and specifically in its receptor binding domain.

VariantHunter makes use of simple statistics and visual representations (line plots and heatmaps) to track viral evolution by highlighting the increase or decrease of prevalence of a given amino acid change or group of amino acid changes (i.e. mutations), for example, those defining a new lineage; analyses are executed by selecting a 4-week time period and an arbitrary geographical area (at the levels of continent, country and region). A ‘dataset explorer’ utility can be used to explore the availability of data and refine the selection of time intervals. Additionally, amino acid changes characteristic of a lineage can be highlighted to facilitate the discovery of novel variants. The tool operates on aggregated data; explicit access to viral genome sequences is not required.

VariantHunter is intended for users that are knowledgeable on the SARS-CoV-2 domain; we address ‘variant hunters’, who are self-professed researchers at the boundary between biology and epidemiology who look for possibly interesting trends to identify and designate the next variant, usually to be communicated on specialized platforms, such as the Pango designation GitHub repository (4) or the Virological forum (5).

In the following, we detail the pipeline that builds the database underlying the VariantHunter tool, we describe its main functionalities, and finally we motivate the use of the application with exemplary use cases gathered from relevant happenings during the COVID-19 pandemic.

## Construction and content

The tool is implemented in the form of a web application, accessible through a web browser. It is composed of a backend (written in Python, using the Flask framework (6)) that executes queries and performs statistical analyses; a frontend (written in Javascript, using the Vue framework (7)) that manages the user input and displays the results; and a text-based database (namely, SQLite3 (8)), where data is loaded.

The current web implementation is based on publicly available genome sequences and associated metadata from GenBank (2), processed according to the *ncov* workflow (9) by Nextstrain (10). The input data file contains one row for each collected sequence. We only consider the columns that contain geo-temporal information, quality information and the list of amino acid changes present for each sequence. Sequences are retained when their length is between 29 000 and 30 000 bases and when the percentage of unknown bases is <0.05.

**Figure 1.**
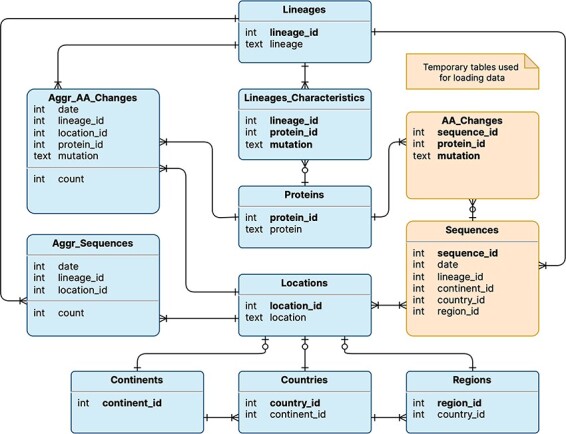
Logical schema of the database used by VariantHunter. Orange tables are created during the parsing of the input file from genomic data sources and then deleted when the blue tables have been constructed, as information about individual sequences is not needed by the tool.

The logical schema of the database is illustrated in Figure 1; this is employed to represent the data model underlying the VariantHunter application. Tables are shown in rectangles with a title (e.g. Lineages) and a list of attributes (e.g. *lineage_id* and *lineage*) with their data types (respectively, integer and text). Table keys are written in bold type. Relationships between tables are expressed with Crow’s foot notation (11): multiplicity in the edge denotes many mappings, a circle on the edge denotes optional mapping and a cut on the edge denotes mandatory mapping. Two tables are built for internal processing, temporarily storing Sequences and their AA_Changes (i.e. mutations on the amino acid level). Persistent tables regard: (i) locations (a geographical area where a viral sequence was collected), represented through three nested levels, i.e. continents that contain countries with regions; (ii) lineages (assigned to sequences with Pangolin (12)), which are characterized by those amino acid changes that appear in at least 50% of sequences assigned to that lineage. The two core tables of the database are Aggr_AA_Changes, i.e. the count of collected sequences (by date, lineage and location) and Aggr_Sequences, i.e. the count of collected sequences with a specific amino acid change (by date, lineage and location). Note that, for each Lineage, it is possible to access the many Aggr_Sequences assigned to it, as well as the Lineage_Characteristics, such as different *protein_ids* (linking to the corresponding protein) and *mutations*. Quantitative information on the database content is provided in Table 1.

The database is well-engineered in order to pre-compute intermediate and aggregate results, according to the design principles that inspired datacubes (13); redundancy is deliberately used to speed up the analysis. The computation of aggregate tables is performed at loading time and the tables storing sequences can be emptied once the computation of aggregate tables is completed. This brings large benefits to the usability of the system: a typical analysis runs in about one second on a common computer. The time required to answer queries (which use as input the date, location and possibly lineage) is independent of both the number of initially loaded sequences and the location level (i.e. continent vs region).

The online system has been running since April 2022 and tested with hundreds of queries on progressively updated datasets, both by the authors and collaborators outside the group. The code is open source and published under Apache License 2.0. To enable the analysis of data with restricted access and/or unpublished data available to users, we also provide a Docker image version of VariantHunter that can be installed on any device supporting the Docker daemon. The installation is optimized for use in combination with the provisioning of input formatted as the GISAID metadata package.

VariantHunter supports two types of analysis on aggregate amino acid change data of collected SARS-CoV-2 sequences: *lineage-independent* and *lineage-specific*. We formalize the sequences available in the system as a set of tuples $S = \,\left\{ {\langle i,d,l,L,M\rangle } \right\}$, where $i$ is an identifier (to ensure uniqueness), $d$ is the collection date, $l$ is the lineage assigned by the most recent release of Pangolin (12), $L$ is the location where the sequence has been collected at a given level of granularity (continent, nation, or region) and $M\, = \left\{ {{m_i}} \right\}$ is a set of amino acid changes. The user interacts with the platform by selecting a date $d^{\prime}$, a specific location $x^{\prime}$ at a given level of granularity, and—in the case of *lineage-specific* analysis—also a lineage $l^{\prime}$.

**Figure 2.**
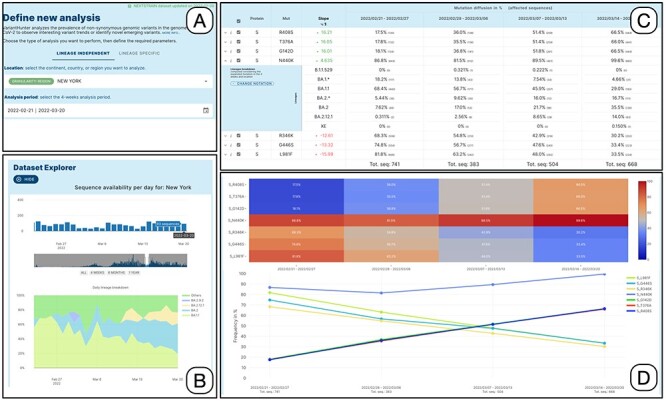
Lineage-independent analysis of sequences collected in the New York state between February 21 and 20 March 2022—corresponding to the transition from Omicron 1 (BA.1) to Omicron 2 (BA.2). Panel A: setting of the analysis parameters; Panel B: overview of the prevalence SARS-CoV-2 lineages; Panel C: amino acid changes with the highest change in prevalence (for the amino acid change N440K on the Spike protein, the lineage breakdown is shown); Panel D: diffusion heatmap and line plot of the prevalence of the changes selected in the table. Three of them have a similar highly increasing trend, N440K is increasing at a slower rate starting from a higher prevalence, whereas the last three are decreasing at various similar rates.

Figure 2 illustrates the interaction process. The user specifies the filters in Panel A, taking advantage of the auto-complete feature in the location field and the calendar drop-down for selecting the date. The choice of the time-window to be analyzed can be supported/refined by the use of the dataset explorer, which provides a bar plot of sequences collected on each date in the selected location. When used in 4-week mode, the explorer also shows a daily breakdown by lineage of the available data (see Panel B).

**Table 1.** Quantitative overview of the current VariantHunter database content

Property	Valu
Date range	23/12/2019–17/04/2023
Number of distinct countries	118
Sequence length range	16 375–32 268 bp
Number of distinct Pango lineages	2521
N. distinct proteins	12
N. distinct AA Changes	58 898
Ten most represented AA Changes	ORF1b: P314L: 7465123; S: D614G: 384484; ORF1a: T3255I: 6134321, S: T478K: 6017739; S: P681H: 4177808; N: R203K: 4159764; N: G204R: 4110908; S: G142D: 4012711; S: N501Y: 3992910; S: H655Y: 3637434

The system then identifies the set of sequences to be analyzed as $S^{\prime}= \{ s \in S:(d^{\prime}- 28 \lt s.d \le d^{\prime}) \wedge \left( {s.L = x^{\prime}} \right)$ $\left[ { \wedge \left( {s.l = l^{\prime}} \right)} \right]\} $, i.e. all the sequences from the user-specified location, (optionally) lineage, and collected in a time frame that spans from 4 weeks before $d^{\prime}$, with $d^{\prime}$ included—note that the lineage selection may be missing depending on the type of analysis. The choice for weekly binning mutated sequences’ counts was motivated by the fact that—usually—countries submit data on a weekly basis, as well as by minimal requirements to generate robust significance and interpretation. Several previous works that study frequency trajectories already made this choice (14–16).

The 4-week period has been chosen as the most appropriate trade-off that captures the weekly periodicity of data collections/submissions and allows extracting amounts of sequences that grant sufficient statistical significance for the trend measurements. One-month periods are typically employed in works that aim to track variants and provide early warning for them (14, 17, 18). The complete set of amino acid changes associated with genome sequences $M^{\prime}= \{ m|\exists s \in S^{\prime}\wedge m \in s.M\} $ is then derived and—for every mutation—levels of prevalence in the first, second, third and fourth week are computed $\left( {{p_1},{p_2},{p_3},{p_4} \in \left[ {0,100} \right]} \right)$.

Detailed results are reported in tabular form as illustrated in Panel C. Each row corresponds to an amino acid change. The *slope* score corresponds to the slope of the regression line for the points $\left\{ {\left( {0,{p_1}} \right),\left( {1,{p_2}} \right),\left( {2,{p_3}} \right),\left( {3,{p_4}} \right)} \right\}$. It is very informative and intuitive, as it does not require statistical knowledge: the higher (lower) the value of the slope, the more the mutation is increasing (decreasing) its prevalence in the target population $S^{\prime}$. The four following columns report the prevalence of the amino acid change at each of the 4 weeks. To assess the statistical significance of prevalence variation, we perform a ${\chi ^2}$ test by comparing, in the four time-points (i.e. weeks): (i) the number of sequences *with* the mutation vs. all the sequences; (ii) the number of sequences *without* the mutation vs. all the sequences; (iii) and the number of sequences *with* the mutation vs. the number of sequences *without* the mutation. *P*-values of each test, adjusted for multiple comparisons, can be optionally displayed (not shown in the panel).

Data on prevalence is represented both as a heatmap and a line plot, as shown in Panel D. By default, the five amino acid changes with the highest rates of increase and decrease are displayed in the charts. However, users are free to select amino acid changes according to their interests, by ticking the related checkboxes. Moreover, users can restrict the set of analyzed mutations by: (i) filtering for a specific protein, (ii) filtering an arbitrary number of table rows, (iii) uploading a list, or (iv) selecting the changes that define a specific lineage (computed as those that are present in at least 50% of the sequences of that lineage) from a dedicated panel.

Each row of the mutation table can be expanded to examine the breakdown of the sequences associated with a specific amino acid change across the different lineages, for each analyzed week. In the figure, the row of the spike amino acid change N440K is expanded showing that the change is predominantly exhibited by sequences assigned to the BA.1.1 lineage (decreasing), whereas its presence in BA.2 sequences is increasing (contributing to the positive trend of the total count of the change). We enable two grouping options for lineages, based on their level in the Pango hierarchy (12). The first option summarizes the counts using a level-1 star notation (e.g. B.1.* includes B.1 and all its descendants). The second option summarizes the counts using a level-2 star notation (e.g. B.1.1.* includes both B.1.1 and all its descendants). In both cases, lineages exceeding 10% (in at least one of the four observed weeks) are not aggregated. Mutations in the table can be further explored, by clicking on the related $i$ symbol. A dialog is opened that informs on two aspects: which lineages are characterized by the selected mutation (i.e. the mutation appears in at least 50% of sequences of those lineages contained in the database) and how the sequences harboring the selected mutation distribute over all available lineages.

The heatmap allows observing the increasing and decreasing trends in a fast way. Two options are available for scaling the plotted color gradients:

- Diffusion (see Figure 2, top of Panel D): colors are scaled between 1 and 100 based on the prevalence of amino acid changes (dark blue = low prevalence and red = high prevalence). This visualization facilitates the identification of highly prevalent amino acid changes.
- Growth (not shown in the figure): colors are scaled based on the ratio between the minimum and maximum prevalence of every amino acid change. This visualization facilitates the identification of amino acid changes that had an increase or decrease in prevalence (dark blue to red = increase and red to dark blue = decrease).

The Diffusion Trend chart plots the same data and lets the users appreciate the point of intersection between the two groups of lineages with opposite trends (in the figure, this happens just before the second week of March 2022). Further characteristics of the trends can be observed using the odd ratio plots. Here, the log2 odd ratio is computed by comparing alternatively the prevalence of each week against the previous one or against the first week of the period. The log2 scale is used to facilitate the comparison of the magnitude of the change between increasing and decreasing amino acid changes.

If needed, an analysis can be easily reproduced with the same settings, changing only the time window, shifting it 1 or 2 weeks either backward or forward. Users can produce several analysis sessions, which are saved in the browser and can be rerun using the ‘history’ sidebar.

**Figure 3.**
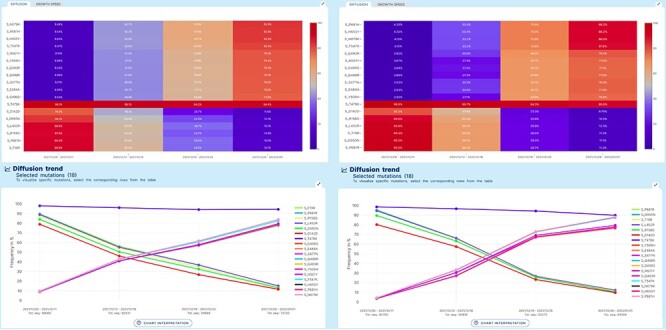
Lineage-independent analysis use case. Spread of the Omicron variant and displacement of Delta in Europe and North America. Note that, for Omicron, only a selection of 11 defining amino acid changes is shown for space constraints.

The analysis of selected mutations can be further assisted by the CoV-Spectrum application, documented in (19). For arbitrarily selected amino acid changes, users can request that a query is generated and submitted to CoV-Spectrum using the same country/continent-level location, time period and set of changes, either in conjunction (logical AND) or in disjunction (logical OR). Once opened, CoV-Spectrum provides additional information about sequences with the specific group of changes. Note, however, that Cov-Spectrum does not provide comparative analysis and prevalence statistics as discussed above (see more information in the Related Work section).

## Utility and discussion

The online documentation http://gmql.eu/variant_hunter/about incorporates a collection of use cases that were selected to illustrate the application of VariantHunter in real-world scenarios. Specifically, they present four examples of lineage-independent analysis to illustrate how VariantHunter could identify the emergence of the ‘paradigmatic’ Alpha, Delta, Omicron 1 and Omicron 4/5 variants. Five examples of lineage-specific analyses are further provided to show the ‘identification’ of mutations with interesting prevalence trends.

In the following, we report one example of both kinds, respectively, representing the emergence of Omicron 1 with the concurrent displacement of Delta and the identification of two mutations with prevalence trends that differ from those of BA.5.2, leading to the recognition of BA.5.2.39.

### Use case of lineage-independent analysis

A ‘lineage-independent’ analysis was applied to study the prevalence of amino acid changes in the Spike glycoprotein in an interval of time spanning from 05/12/2021 to 01/01/2022 in Europe and in North America. This interval coincides with the rapid emergence and spread of the Omicron VOC of SARS-CoV-2 worldwide.

Figure 3 represents the results produced by running an analysis on sequences collected in Europe data (left) or in North America (right). Both diffusion heatmaps and trend charts show a clear decrease in the prevalence of six amino acid changes characteristic of the Delta VOC (G142D, D950N, L452R, R158G, P681R and T19R) and a concomitant and striking increase of amino acid changes characteristic of the Omicron VOC* of SARS-CoV-2. Interestingly, the prevalence of T478K, an amino acid change shared by both VOCs, is only marginally affected. From the comparison of left and right plots, we can appreciate that Europe has observed the switch between the two variants slightly earlier than North America.

### Use case of lineage-specific analysis

Currently, significant manual effort is required in designating new lineages in the present system. First, volunteers collect and present evidence of a possible novel lineage that they observed; this is communicated through the Pango designation Github platform (4) by opening a new issue. Then, domain experts carefully review the proposals for potential errors, before formally designating the new lineage. This process requires time and does not follow any declared systematic methodology.

**Figure 4.**
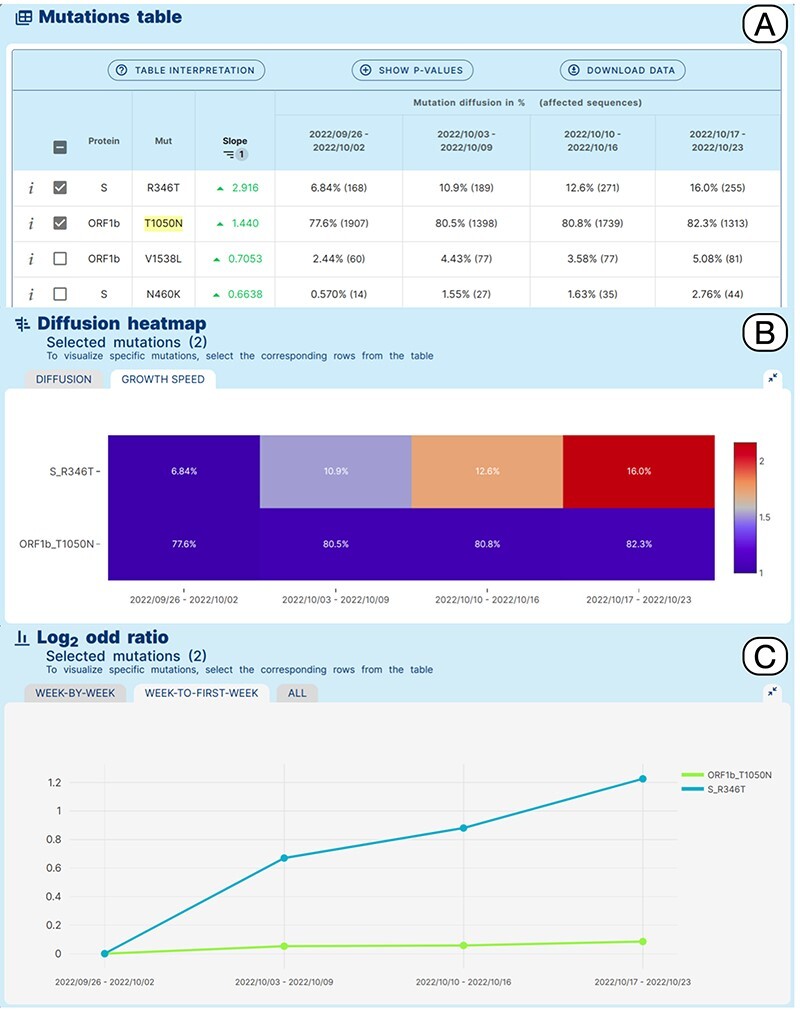
Lineage-specific analysis use case. Emergence of sub-lineage BA.5.2.39 in North America.

VariantHunter can provide data-driven support to this process, as described in the following example. BA.5.2.39, a sublineage of BA.5.2 that carries an additional R346T amino acid change in the Spike glycoprotein compared with its ancestor, was designated by the Pango issue 1279 (https://github.com/cov-lineages/pango-designation/issues/1279). According to the submitter of the issue, BA.5.2.39 derives from an un-named/un-designated sub-lineage of BA.5.2 characterized by the presence of a T1050N amino acid change in ORF1b. Interestingly this amino acid change was observed in more than 50% of the genomic sequences assigned to BA.5.2 at a later time (beginning of December 2022).

A Variant Hunter ‘lineage-specific’ analysis was applied to study amino acid changes in the BA.5.2 lineage, in North America in an interval of time between 26/09/2022 and 23/10/2022, which should coincide with the emergence and spread of BA.5.2.39. Figure 4 shows the obtained results. The Mutation Table (Panel A) shows a clear increase in the prevalence of Spike R346T in sequences assigned to BA.5.2 (6.8%–16%) in the selected interval of time. Interestingly, a more moderate relative increase of ORF1b_T1050N from 77.6% to 82.3% is also observed. This pattern is clearly illustrated by the growth speed diffusion heatmap (Panel B) and by the matched Log2 odd ratio diffusion plot (Panel C), where a more than 2-fold increase in the prevalence of S_R346T is recovered.

## Related work

Other systems have been proposed in the past 2 years for exploring the evolution of known SARS-CoV-2 lineages. COVID-19 CG (20) tracks SARS-CoV-2 mutations and lineages with filters available on location, date, gene, or mutation of interest. It is a utility that allows highly interactive searches, but does not provide automatic aggregation or statistical testing. Outbreak.info (21) produces informative reports on known lineages—possibly combined with single amino acid-level mutations. Mutations need to be specified by users, as they are not ranked by the system. It is useful for getting insights into the evolution of the virus SARS-CoV-2 and as a hypothesis generation tool; its focus is not on mutations whose patterns shift from others. We previously built ViruClust (22), a tool for comparing SARS-CoV-2 genomic sequences and lineages in space and time. ViruClust allows to build two custom sets of sequences (based on spatio-temporal or lineage-based filters), denoted as ‘target’ and ‘background’ and to compare the pair-wise prevalence of their common amino acid changes. Differently, VariantHunter, introduces the novelty of trend detection over different points in time, prioritizing highly increasing or decreasing amino acid changes; computations are very fast as the database is pre-computed and optimized for 4 weeks observations.

CoV-Spectrum (19) supports both the tracking of known variants and the identification of new SARS-CoV-2 variants of concern. It presents a flexible search interface that allows building filters as a conjunction or disjunction of single amino acid/nucleotide mutations. It proposes the use of several computational methods to gather the proportion of different variants over time, their demographic and geographic distributions, probabilities of hospitalization and mortality, as well as estimates for transmission fitness advantage (23). CoV-Spectrum can be considered a variant-centric resource, whose finest granularity level reaches countries. Differently, VariantHunter is mutation-centric: for a given period and a given location (including also regions), it provides an overview of single mutation trends, observed independently from (or in disagreement with) a lineage. The system allows the prioritization of specific sets of mutations, drawing attention to their prevalence, increase, or decrease trends. Once the user has acquired this information, further detailed analysis—based on specific mutation patterns appearing on single genomes—can be performed on other existing systems. To promote this possibility, VariantHunter already includes a direct call from its tabular results to the CoV-Spectrum system.

## Conclusions

A systematic and well-assisted understanding of prevalence changes of lineages and of their specific mutations is the fundamental ingredient of viral genomic surveillance. VariantHunter does not aim to replace phylogenetics methods, which are fundamental to eventually identifying new lineages and their determination. What VariantHunter provides, instead, is a tool to suggest suitable directions to look for new variants. As the search space for such variants is very large (considering all the locations, periods of time, lineages and mutations that single sequences can acquire), a tool such as VariantHunter allows to speed the work of researchers when assessing groups of sequences with mutations that are growing particularly fast.

VariantHunter is proposed as a utility that allows significant savings in time, labor and costs for projects on genomic surveillance of SARS-CoV-2; it provides a user-friendly method for querying the changes in prevalence for mutations in a given location over a given period of 4 weeks of time.

The web service is designed to be as easy to use as possible and also responsive for answering user-generated hypotheses, with a reactive interface. The time required by data analysis is very short and does not depend on either the size of the initial dataset or the specific kind of analysis, as data aggregations are computed at loading time.

VariantHunter’s simple paradigm could be reapplied to any kind of dataset with similar characteristics, also in the context of possible future pandemics. This possibility is empowered by a well-documented Docker version of the system that is distributed along with the open online web service.

## Data Availability

The website and documentation are available at http://gmql.eu/variant_hunter/. The software code is provided at https://github.com/DEIB-GECO/VariantHunter/ under the license Apache License 2.0.
